# PE11 (Rv1169c) selectively alters fatty acid components of *Mycobacterium smegmatis* and host cell interleukin-6 level accompanied with cell death

**DOI:** 10.3389/fmicb.2015.00613

**Published:** 2015-06-23

**Authors:** Wanyan Deng, Jie Zeng, Xiaohong Xiang, Ping Li, Jianping Xie

**Affiliations:** State Key Laboratory Breeding Base of Eco-Environment and Bio-Resource of the Three Gorges Area, Key Laboratory of Eco-environments in Three Gorges Reservoir Region, Ministry of Education, School of Life Sciences, Institute of Modern Biopharmaceuticals, Southwest UniversityChongqing, China

**Keywords:** *Mycobacterium tuberculosis*, PE family, Rv1169c, fatty acid components, cell wall integrality, cell death, interleukin-6

## Abstract

PE/PPE family proteins, named after their conserved PE (Pro-Glu) and PPE (Pro-Pro-Glu) domains of N-terminal, are most intriguing aspects of pathologic mycobacterial genome. The roles of most members of this family remain unknown, although selected genes of this family are related to the virulence of *Mycobacterium tuberculosis*. In order to decipher the role of Rv1169c, the *Mycobacterium smegmatis* strain heterologous expressed this ORF was constructed and identified that Rv1169c was a cell wall associated protein with a novel function in modifying the cell wall fatty acids. The growth of Rv1169c expressing strain was affected under surface stress, acidic condition and antibiotics treatment. *M. smegmatis* expressing Rv1169c induced necrotic cell death of macrophage after infection and significantly decreased interlukin-6 production compared to controls. In general, these results underscore a proposing role of Rv1169c in virulence of *M. tuberculosis*, as it's role in the susceptibility of anti-mycobacteria factors caused by modified cell wall fatty acid, and the induced necrotic cell death by Rv1169c is crucial for *M. tuberculosis* virulence during infection.

## Introduction

Tuberculosis (TB), caused by *Mycobacterium tuberculosis* infection, remains a formidable threat to global public health. *M. tuberculosis* can modulate and elude host immune responses and persist for prolonged periods (Lin and Flynn, 2010). One hallmark of the *M. tuberculosis* genome is the presence of the multi-genic PE/PPE family proteins, which consist of PPE, PE, and PE_PGRS subfamilies and account for about 10% of the coding capacity of the *M. tuberculosis* genome. There are 69 genes encoding PPE subfamily which are named after its N terminal Pro(P)-Pro(P)-Glu(E) motif (Cole and Barrell, 1998; Cole et al., 1998) and about 100 genes encoding PE subfamily, which harbor a conserved N-terminal domain with 110 amino acid residues with the Pro-Glu (PE) motif, while the C-termini vary significantly in size and protein-specific polymorphic GC-rich repeats PGRS (Cole et al., 1998; Akhter et al., 2012). The 69 PPE proteins are classified into PPE_SVP with typical G-X-S-V-P-X-X-W repeats, PPE_PPW with special G-F-X-G-T and Pro-X-X-P-X-X-W sequences and PPE_MPTR with N-X-G-X-G-N-A-G major polymorphic tandem motifs in their C terminal (Cole and Barrell, 1998; Cole et al., 1998). The PE subfamily contains 37 PE genes with a conserved N terminal and 61 PE_PGRS genes with G-G-A and G-G-N tandem repeats in their C terminal (Brennan and Delogu, 2002; Fleischmann et al., 2002; Voskuil et al., 2004; Dheenadhayalan et al., 2006a). The unique sequences of these proteins might underlie the specific physiological role of this family during *M. tuberculosis* infection.

The exclusive presence of PE/PPE family among pathogenic mycobacteria (Gey van Pittius et al., 2006) has attracted many researchers. The primary origin, regulation and physiological role of some PE/PPE family proteins have been well characterized and reviewed (Brennan and Delogu, 2002; Tian and Jian-Ping, 2010; Mohareer et al., 2011; Sampson, 2011; Akhter et al., 2012; Kohli et al., 2012; Vordermeier et al., 2012; Fishbein et al., 2015). The origin of the PE/PPE genes is associated with the Type VII secretion system (T7S) (Abdallah et al., 2009). Several transcriptional regulators involved in the regulation of PE/PPE family proteins have been characterized, including the stringent response mediator RelA (Dahl et al., 2003), ESX-1 secreted protein regulator EspR (Blasco et al., 2012) and global nucleoid-associated transcriptional inhibitor Lsr2 (Gordon et al., 2010) and sigma factors, such as sigF (Williams et al., 2007; Humpel et al., 2010), sigB (Dahl et al., 2003; Fontan et al., 2009), and sigD (Raman et al., 2004; Calamita et al., 2005). It has been shown that several members of PE/PPE proteins are immunogenic (Delogu and Brennan, 2001; Chaitra et al., 2005; Campuzano et al., 2007) and might contribute to the antigenic diversity and immune evasion of mycobacteria (Cole et al., 1998; Brennan and Delogu, 2002). The C-terminal fragments of the PE_PGRS protein Rv1759c (Espitia et al., 1999) or the PGRS domain of Rv3367 (Singh et al., 2001) can react with TB patients sera. Other PE/PPE proteins such as PE25/PPE41 complex, Rv1169c, Rv0978c as well as Rv1818c showed same characteristics (Delogu and Brennan, 2001; Narayana et al., 2007; Tundup et al., 2008). Some PE/PPE proteins are implicated in immune evasion and antigenic variation (Banu et al., 2002; Brennan and Delogu, 2002) or may be linked to virulence and responsible for its ability to grow in a macrophage (Jha et al., 2010; Dong et al., 2012; Iantomasi et al., 2012; Tiwari et al., 2012; Thi et al., 2013). PE/PPE family proteins are cell wall associated (Delogu et al., 2004; Cascioferro et al., 2007, 2011; Dona et al., 2013; Chatrath et al., 2014; Deng et al., 2014), suggesting that a role in directly interaction with host targets such as the cell surface receptor TLR2, or even interfering the host immunity (Nair et al., 2009; Bansal et al., 2010; Tiwari et al., 2012; Zumbo et al., 2013; Deng et al., 2014). Macrophages are the first line of defense against bacteria infection, which can secrete various cytokines to mediate the inflammatory response. The varied transcription level of several PE/PPE genes within macrophages or mouse during *M. tuberculosis* infection (Dheenadhayalan et al., 2006b) suggested a role in manipulation the host macrophage activity. PE_PGRS62 inhibited the maturation of phagosome (Huang et al., 2012), PE_PGRS30 (Iantomasi et al., 2012), as well as PPE25 (Jha et al., 2010) disturbed the phagolysosomal fusion. In brief, mounting evidences showed that PE/PPE family proteins contribute significantly to the successful immune evasion of *M. tuberculosis*.

*M. tuberculosis* Rv1169c encodes for PE11, a prototypical member of PE/PPE family protein, was characterized in this study. The up-regulation of Rv1169c after 24 h of starvation (Betts et al., 2002) or transient acid exposure (Fisher et al., 2002), suggested a role in pathogen persistence or dormancy, in particular, in fatty acid metabolism. To study the role of Rv1169c and underlying mechanism, *M. smegmatis* expressing Rv1169c and *M. smegmatis* harboring the vector only were constructed. Our studies demonstrated that *M. smegmatis* expressing Rv1169c has a modified cell wall fatty acid consonant, and its ability to against anti-tuberculosis factors was abated. Moreover, Rv1169c was also found to affect the pleiotropic pro-inflammatory cytokine IL-6 secretion and manuscript cell death of macrophage.

## Methods and materials

### Construction recombinant *M. smegmatis* strains

Mycobacterial expression vector pALACE used in this study has been described previously (Lakshminarayan et al., 2008). The full-length of Rv1169c gene was amplified from *M. tuberculosis* genome using gene-specific primers listed in Table 1 (pALACE-Rv1169c-F and pALACE-Rv1169c-R). The *Eco*R I-*Bam*H I-digested PCR product was cloned into pALACE to generate pALACE-Rv1169c. The plasmids (pALACE and pALACE-Rv1169c) were electroporated into fast-growing non-pathogenic *M. smegmatis* mc*^2^*155 according standard procure (Lakshminarayan et al., 2008). The recombinan*t M. smegmatis* strains were selected on MB 7H10 medium containing 100 μg/ml hygromycin (Hyg). The constructs harboring Rv1169c gene were confirmed by PCR amplification, and the positive recombinant strains were stored with sterile 20% glycerol at −80°C for further use. *Escherichia coli* DH5a strains using for gene cloning were grown at 37°C using Luria–Bertani (LB) broth and LB agar with the addition of appropriate antibiotics. *M. smegmatis* mc^2^155 were grown in 7H9 broth medium or on 7H10 agar supplemented with 0.05% (v/v) Tween 80, 0.2% (w/v) glucose, and 0.5% (v/v) glycerol.

**Table 1:** **Primers used in this study**.

**Primers**	**Sequence (5′-3′)**
pALACE-Rv1169c-F	CCGGAATTCGTGTCTTTTGTCA
pALACE-Rv1169c-R	CGGGATTCGTAGGTGGAGGT
TNF-α-F	GGCGGTGCTTGTTCCT
TNF-α-R	GCTACAGGCTTGTCACTCG
IL-6-F	GCCTTCGGTCCAGTTGCCTTCT
IL-6-R	TGCCAGTGCCTCTTTGCTGCTTT
IL-1β-F	TTCAGGCAGGCCGCGTCAGTTGT
IL-1β-R	TGTGAGTCCCGGAGCGTGCAGTT
IL-10-F	ACCTGGGTTGCCAAGCCTTGT
IL10-R	GCTCCACGGCCTTGCTCTTGTTT
IL-12p40-F	CATCATCAAACCTGACCCACC
IL12p40-R	CTTTTCTCTCTTGCTCTTGCCC
Casepase-1-F	GAAGGTACAATAAATGGCTTAC
Casepase-1-R	GAATAACGGAGTCAATCAAA
Bcl2-F	TGCTGTGGCTTCTGTG
Bcl2-R	GGGCTGGATTTCTCAA
β-action-F	GTGACGTTGACATCCGTAAAGA
β-action-R	TGTGAGTCCCGGAGCGTGCAGTT

### Detection the expression of Rv1169c in *M. smegmatis*

The recombinant *M. smegmatis* strains harboring His-tagged Rv1169c (Ms_Rv1169c) and vector pALACE (Ms_Vec) were cultured in MB 7H9 broth medium supplemented with 100 μg/ml Hyg. At the *OD*_600_-value of 0.8, the recombinant strains were subjected to 28 mM acetamide (Aladdin, China) for protein expression. *M. smegmatis* cell fractionation was carried out essentially as described earlier, with minor modifications. In general, the recombinants including Ms_Vec and Ms_Rv1169c were harvested after 16 h acetamide induction using centrifugation at the speed of 3000 × g for 10 min, 4°C. The collected cells were washed and then sonicated in cold PBS supplemented with protease inhibitor P-8849 (Sigma-Aldrich). After sonication, the prepared whole-cell lysate were centrifuged at the speed of 20,000 × g for separating the insoluble (pellets in the bottom) and the soluble (supernatant in the upper layer) fractions. The separated fractions were loaded to SDS-PAGE and further detected by Western blot analysis with using specific anti-His monoclonal antibody (TIANGEN, China). The blots were formed when incubation with IgG-HRP, an anti-mouse IgG monoclonal antibody labeled with horseradish peroxidase (TIANGEN, China).

### Subcellular fractionation of recombinant *M. smegmatis*

Recombinant Ms_Rv1169c and Ms_Vec constructs were grown and subjected to cell fractionation separation as described previously (Deng et al., 2014), with minor modification. Generally, the acetamide-induced recombinant Ms_Vec and Ms_Rv1169c were subjected to sonication. The whole lysates were centrifuged at the speed of 3000 × g for 5 min at 4°C for removing un-lysed cells and cell debris. The supernatants were ultra-centrifuged at the speed of 27,000 × g for 30 min, at 4°C. After ultra-centrifugation, the pellets were considered the cell wall fraction, and the supernatants were supposed to cell membrane and cytosol fractions. The pellets were further suspended in PBS. Equal amounts of protein from pellets and supernatants fraction were subjected to Western blot as described previously for analysis the expression of Rv1169c.GroEL2 protein served as cytosol marker protein of mycobacteria.

### Analysis *in vitro* survival under different stress conditions

Recombinant *M. smegmatis* strains were grown into optimal concentration in 7H9 medium containing 100 μg/ml Hyg. Ms_Vec and Ms_Rv1169c were performed in presence of stress condition after 16 h induction by acetamide. Ms_Vec and Ms_Rv1169c were treated by 0.05% SDS for 1, 2, 3, and 4 h. In addition, pH gradient was generated by adding HCl into 7H9 medium, and sterilized by passing through a 2 μm filter. After the treatment, the recombinant strains were ten-fold dilution spotted onto MB 7H10 agar containing Hyg and bacteria numbers were counted after 3 days.

### Anti-tuberculosis drug sensitivity assays

Four antibiotics were used in this study, including vancomycin (Van), isoniazid (INH), norfloxacin (Nor) and rifampicin (Rif). Acetamide induced Ms_Vec and Ms_Rv1169c strains were prepared for treatment with these four different antibiotics. The original concentration for Van is 320, 512 ug/ml for INH, 512 ug/m for Nor and 128 ug/m for Rif, and different concentration of each antibiotic was made prepared by 2-fold dilution. MIC values of each antibiotic were determined when the bacterial activity was killed at least 99% on liquid medium. For bactericidal ability test, Acetamide induced Ms_Vec and Ms_Rv1169c strains were exposed to Van (5, 10, 20, 80, 160 μg/ml), INH (4, 16, 64, 128 μg/ml), Nor (1, 2, 4, 8, 32, 64 μg/ml) and Rif (4, 16, 64, 128 μg/ml) for 24 h, respectively. After treatment with these four antibiotics in different concentration, the recombinant strains were diluted by 10-fold and plated into 7H10 agar medium and counted the bacterial number after 3 days culture. The medium without any antibiotics serves as the control to make sure the normal growth of bacteria.

### Macrophages infection by recombinant *M. smegmatis*

The human monocyte cell line U-937 was maintained in RPMI 1640 medium (Invitrogen) supplemented with 2 mM L-glutamine, 10% (v/v) heat inactivated FBS, 100 U/ml penicillin and 100 μg/ml streptomycin (Invitrogen) and cultured in humidified incubator supplemented with 5% CO_2_ at in 37°C. Macrophages were seeded at 1 × 10^6^ cells per well in 12-well tissue culture plates or at 5 × 10^5^ cells per well in 24-well tissue culture plates. The suspension cell line U-937 cells were transformed into adherent macrophage after 48 h treatment with 100 μg/ml phorbol 12-myristate 13-acetate (PMA, Sigma). Cells were infected with Ms_Vec or Ms_Rv1169c at MOI of 10. Gentamicin was added into culture at the concentration of 150 ug/ml after 4 h infection to remove the bacteria outside the macrophages. After 6, 24, 48, and 72 h infection, the culture supernatants were collected for detecting lactate dehydrogenase (LDH) activities. The LDH activities were detected by commercially LDH cytotoxicity kit (Takara Bio) according to standard procedure. For the detection of bacteria survival within macrophage, the infected macrophages were washed by PBS for 3 times and lysed by 0.05% SDS. The cell lysates were serially ten-fold diluted and then spotted on 7H10 agar containing Hyg. The numbers of bacteria were enumerated after 3 days.

### Assay for cytokines production

Culture supernatants were harvested after infection of macrophages with Ms_Vec or Ms_Rv1169c for 6, 24, 48, and 72 h. The RNA was extracted from the infected cells using RNA extraction kit (TIANGEN) and mRNA level of cytokines were detected by RT-PCR using gene specific PCR primers listed in Table 1. The cytokines in the culture supernatants were detected using commercially ELISA kits of interleukin 1β(IL-1β), interleukin 6 (IL-6).

### GC-MS analysis of fatty acid components

The samples used in GC-MS analysis were extracted according the standard procedure (http://www.midi-inc.com). The determination of fatty acids was performed on an Agilent 7890A gas chromatograph with GC-MSD (5975C mass selective detector) equipped with an Agilent 7693A automatic liquid sampler and a DB-5MS capillary column (30 m, 0.25 mm i.d, 0.25 μm film thickness). High purity (99.999%) helium served as the carrier gas in constant-flow mode at a column flow rate of 1 mL/min. 150, 230, 250, and 280°C were the optimal temperature of quadrupole, ion source, injector and transfer line temperatures, respectively. The oven temperature program was as follows: initial temperature 60°C, held for 1 min; increased to 160°C; later increased to 290°C held for 10 min. 70eV was set for the electron impact energy. One microliter of each sample was infused in splitless mode. PAHs were identified by comparing the relative RT (retention time) with IS. Those peaks were integrated for quantification and qualification when their location was within the right range (2%) of RT.

### Statistical analysis

Graphpad Prism 6 software was used for analysis of differences between experimental and control group. Statistical significance (*P*-value) was decided using Student's *t*-test. *P*-values were less than 0.05 were supposed to be statistically significant. ^*^P < 0.05, ^**^P < 0.01, and ^***^P < 0.001.

## Results

### *M. tuberculosis* Rv1169c can be expressed in *M. smegmatis*

**Figure 1:**
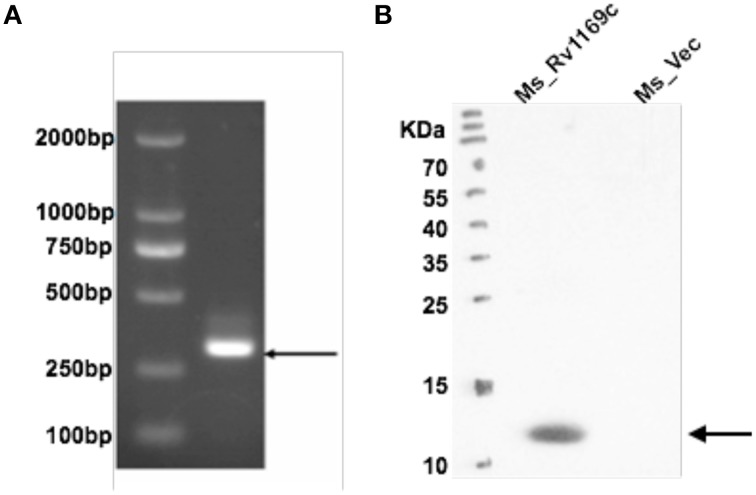
**Heterologous expression of** ***M. tuberculosis*** **Rv1169c in** ***M. smegmatis***. **(A)** Ms_Vec and Ms_Rv1169c were grown into an OD_600_ of 0.8 and then were subjected to PCR amplification to detect Rv1169c gene. **(B)** The cell lysates were prepared from acetamide-induced Ms_Vec and Ms_Rv1169c were subjected to Western blot to determine the expression of His-tagged Rv1169c protein in *M. smegmatis* by mouse anti-His antibody.

*M. tuberculosis* PE/PPE family Rv1169c gene encodes 12 kDa protein with about 300 bp in size. In this study, we constructed two recombinant *M. smegmatis* strains to probe the role of Rv1169c, recombinant Ms_Vec and Ms_Rv1169c strains were constructed. Ms_Rv1169c expressed a His-tagged Rv1169c protein harboring pALACE-Rv1169c, while Ms_Vec only harbored the vector with Hyg resistant marker gene (Lakshminarayan et al., 2008). Both Ms_Vec and Ms-Rv1169c were grown in MB 7H9 medium supplemented with Hyg. PCR amplification identified there was about 300 bp of Rv1169c gene in Ms_Rv1169c strain by specific primers (Figure 1A). Western Blot confirmed only Ms_ Rv1169c strain expressed ~12 kDa Rv1169c-His protein, while its absence in Ms_Vec strain (Figure 1B). These results identify that *M. tuberculosis* Rv1169c gene was constructed and expressed in *M. smegmatis* strains.

### Rv1169c is associated with mycobacterial cell wall

**Figure 2:**
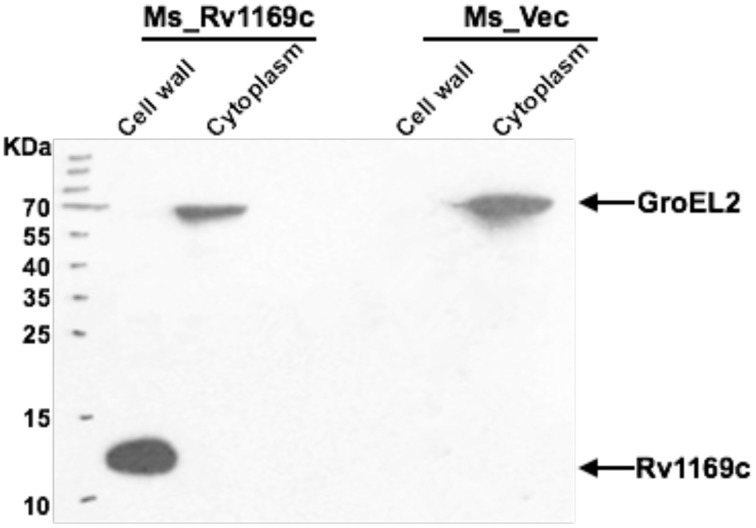
**Rv1169c is associated with** ***Mycobacterium*** **cell wall**. Cell fractionation experiments were performed to detect detection the expression of Rv1169c protein, and further confirmed by Western blot by specific anti-His antibody. The cytoplasm marker of *Mycobacterium* GroEL2 served as a control.

Bioinformatics approach suggested that at least 29 PE/PPE proteins are associated with the “cell wall and cell processes” (Mazandu and Mulder, 2012). Selected PE/PPE genes were experimentally identified localized to cell wall of mycobacteria (Fishbein et al., 2015). We hypothesized that Rv1169c might be also localized to the cell wall of *M. smegmatis*. In order to further identify this hypothesis, *M. smegmatis* expression His-tagged Rv1169c strain was constructed. Recombinant *M. smegmatis* strains including Ms_Rv1169c and Ms_Vec were subjected to cell fractionation experiment, their subcellular localization was finally determined by Western blot. The Rv1169c protein was present in the cell wall fraction and was not found in cytoplasm fraction (Figure 2), suggesting Rv1169c is a cell wall-associated protein. As expected, cytoplasmic heat-shock protein GroEL2 was detected only in the cytoplasm of *M. smegmatis*.

### Rv1169c reprograms the cell wall components

**Figure 3:**
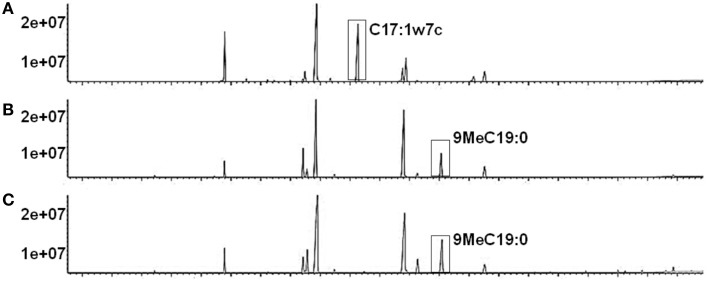
**The fatty acid component in cell wall of recombinant Ms_Rv1169c (A), Ms_Vec (B), and wild-type** ***M. smegmatis (C)***.

The cell wall associated Rv1169c protein would provide a proof for the observed phenotypes related to the characteristic of cell surface, as another PE/PPE member PE30 (also designated lipase LipY) involved in lipids metabolism and was mainly present in the cell wall (Mishra et al., 2008). Rv1169c was predicted as lipase LipX (Deb et al., 2006). We therefore tested whether Rv1169c was involved in lipids metabolism, recombinant Ms_Rv1169c, Ms_Vec and wild type *M. smegmatis* strains were subjected to GC-MS to determine the fatty acids components of these strains. A novel unsaturated fatty acid C17: 1w7c was found in the cell wall component of recombinant Ms_Rv1169c while another saturated 9MeC19:0 was disappeared compared with Ms_Vec and wild type *M. smegmatis* strains (Figure 3). The difference of saturability of fatty acid between Ms_Rv1169c and wild type *M. smegmatis* might be due to the directly and indirectly effect of Rv1169c on the expression of *Mycobacterium* fatty acid desaturase. On the other hand, the modified components in cell wall of Ms_Rv1169c may affect their permeability to antimicrobial factors and their survival in the host.

### Ms_Rv1169c is highly sensitive to anti-tuberculosis drugs

**Figure 4:**
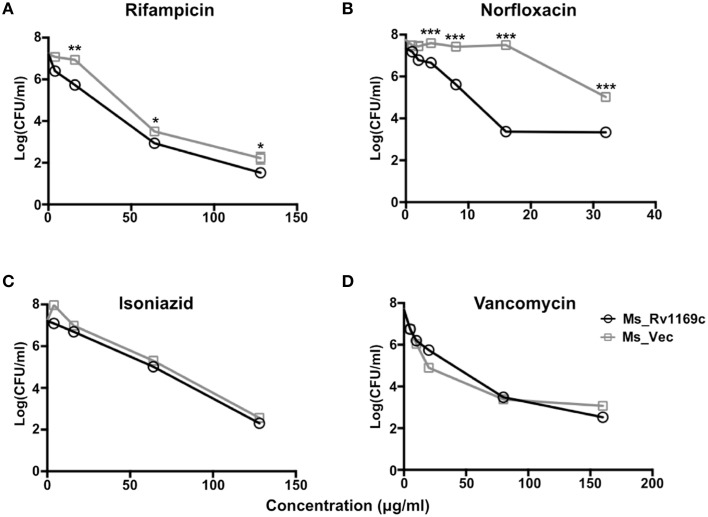
**The survival of Ms_Vec and Ms_Rv1169c after treatment with different antibiotics, including Rif (A), Nor (B), INH (C), and Van (D)**. Ms_Vec and Ms_Rv1169c were exposed to Rif, Nor, INH and Van, and then ten-fold dilution spotted the bacteria on MB 7H10 supplemented with Hyg, the bacterial number of Ms_Rv1169c and Ms_Vec were counted after 3 days cultivation. ^*^ *P* < 0.05, ^**^ *P* < 0.01, and ^***^ *P* < 0.001.

The cell wall serves as an effective permeable barrier of *M. tuberculosis* for diverse antibiotics (Brennan and Nikaido, 1995). Therefore, the alterations of cell wall fatty acid component in Ms_Rv1169c might be highlighted a role of Rv1169c in cell wall permeability. To study whether Rv1169c function in cell wall permeability, Ms_Vec and Ms_Rv1169c were treated with four anti-tuberculosis drugs as described in method. Both Ms_Vec and Ms_Rv1169c displayed comparable capacity to Van and INH. The MIC values of Van and INH for Ms_Vec were 5 and 1 μg/ml, and 2.5 and 0.5 μg/ml for Ms_Rv1169c, respectively. However, Ms_Rv1169c was highly susceptible to Rif and especially sensitive to Nor. The MIC values of Rif and Nor for Ms_Vec were 8 and 16 μg/ml, while 2 and 1 μg/ml for Ms_Rv1169c, respectively. The MIC values of Ms_Rv1169c for Rif and Nor were 4 and 16 fold lower than Ms_Vec, respectively (Table 2). In addition, the Ms_Rv1169c was more sensitive than Ms_Vec to Rif, especially Nor, while there was no difference in INH and Van (Figure 4). These results thus suggest a novel function of PE family protein Rv1169c in the susceptibility of antibiotic.

**Table 2:** **Drug sensitivity to anti-tuberculosis drugs**.

**Anti-tuberculosis drugs (μg/ml)**	**Ms_Vec**	**Ms_Rv1169c**
Van	5	2.5
INH	1	0.5
Rif	8	2
Nor	16	1

### Ms_Rv1169c is more susceptible to antimicrobial factors

**Figure 5:**
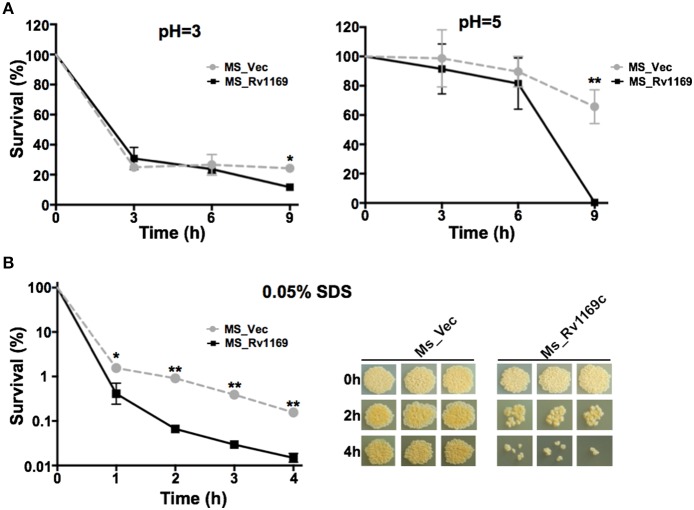
**The sensitivity of Ms_Vec and Ms_1169c strains to acid and surface stress. (A)** *In vitro* growth of recombinant Ms_Vec and Ms_Rv1169c after treatment with different pH gradient for 0, 3, 6, and 9 h. The Ms_Vec and Ms_Rv1169c strains were centrifuged, re-suspended to 5 ml MB 7H9 at an OD_600_ of 0.5, 10-fold serial dilutions of Ms_Vec and Ms_Rv1169c were spotted on MB 7H10 containing Hyg. **(B)** Survival of Ms_Vec and Ms_Rv1169c after exposure to 0.05% SDS. Re-suspended 5 ml recombinant MS_Vec and MS_Rv1169c (*OD*_600_ = 0.5) were exposed to 0.05% SDS for 1, 2, 3, and 4 h. And then the recombinant strains were plated onto 7H10 plates by serially ten-fold dilution, the bacterial numbers were counted after 3–4 days of cultivation at 37°C. ^*^ *P* < 0.05 and ^**^ P < 0.01.

To gain further insight into whether Rv1169c will affect the permeability to antimicrobial factors, the growth characteristics of recombinant Ms_Vec and Ms_Rv1169c under different acid condition and surface stress were analyzed. As shown in the Figure 5A, there was no significant difference between Ms_Vec and Ms_Rv1169c under *in vitro* acid stress at early time points (0, 3, 6 h after treatment). The survival percentage of Ms_Rv1169c was significant lower than Ms_Vec after 9 h treatment with acid stress (pH = 3 and 5). The acid sensitive mutants of *M. tuberculosis* were observed also hypersensitive to antibiotics, surface stress, heat shock, oxidative stress, oxygen and nitrogen intermediates (Vandal et al., 2009). Whether Ms_Rv1169c was hypersensitive to surface stress, Ms_Vec and Ms_Rv1169c were exposed to 0.05% SDS that mimicked surface stress. Although there was a rapid decrease in the bacterial numbers for all tested strains exposed to the detergent SDS (Figure 5B), Ms_Rv1169c was more sensitive to SDS compared to Ms_Vec: the survival percentage was 0.5% for Ms_Rv1169c while 3% for Ms_Vec. The sensitivity of Ms_Rv1169c to SDS was validated when bacterial survival was tested after 1 h of incubation with SDS (Figure 5B). These results suggest that Rv1169c promotes the susceptibility of *M. smegmatis* to surface and acid stress.

### Intracellular survival of recombinant *M. smegmatis* within macrophages

**Figure 6:**
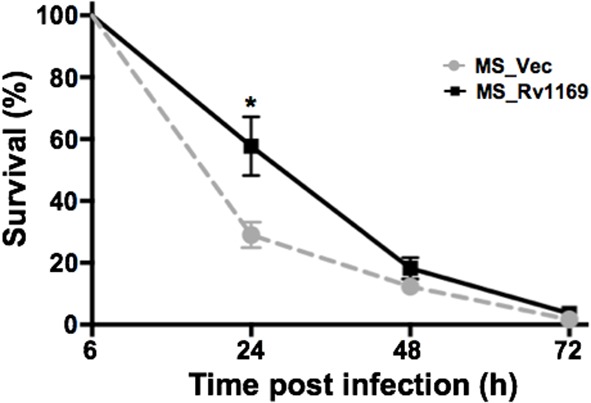
**Intracellular survival of recombinant Ms_Vec and Ms_Rv1169c within macrophages**. PMA-differentiated macrophages were infected with Ms_Vec or Ms_Rv1169c at an MOI of 10. At 6, 24, 48, and 72 h after infection, the macrophages were washed and lysed using 0.01% SDS. Lysates were plated on MB 7H10 medium containing 100 μg/ml Hyg to determine the bacterial number. ^*^ *P* < 0.05.

Several PE/PPE proteins are responsible for the virulence *M. tuberculosis* and contribute to its ability to grow in a macrophage (Bottai et al., 2012; Tiwari et al., 2012; Singh et al., 2013). Whether Rv1169c will affect the intrecellur survival of *M. smegmatis* in host macrophage, as selected PE/PPE proteins were reported to mediate bacterial survival within macrophage through different mechanisms (Tiwari et al., 2012; Singh et al., 2013). We compared the survival rate of recombinant Ms_Rv1169c and Ms_Vec to determine whether Rv1169c can enhance the intracellular survival of *M. smegmatis* within macrophages. PMA-differentiated U-937 macrophages were infected with Ms_Vec and MS_Rv1169c at an MOI of 10. There was a significant difference in the percent survival of bacteria in macrophages between Ms_Rv1169c and Ms_Vec at 24 h after infection (Figure 6), implying the presence of Rv1169c can enhance the intracellular survival of *M. smegmatis* within macrophages at early stage of infection.

### Ms_Rv1169c promotes the death of macrophage

**Figure 7:**
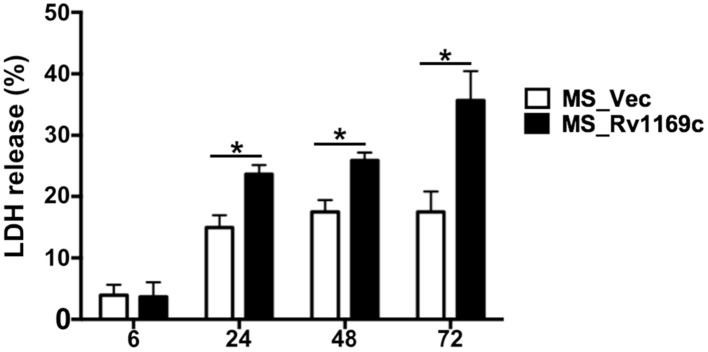
**Assay of cell death in macrophages infected with Ms_Vec or Ms_Rv1169c**. Macrophages were infected with Ms_Vec (white bars) or Ms_Rv1169c (black bars) at an MOI of 10. After 6, 24, 48, and 72 h infection, culture supernatants were collected and the release of LDH was measured. Data are shown as means ± SEM of triplicate wells. ^*^ *P* < 0.05.

Infection of macrophages with *M. tuberculosis* can induce necrosis, defined by cell lysis. *M. tuberculosis* might manipulate host cell death to cause disease. Alternatively, infection can result in macrophages apoptosis to maintain an intact plasma membrane, to diminish pathogen viability and enhance host immunity. To determine the effect of Rv1169c on viability of macrophages, PMA-differentiated macrophages were infected with Ms_Vec or Ms_Rv1169c, LDH release into the culture supernatants was determined after Ms_Vec and Ms_Rv1169c infection. LDH release from infected macrophage was increased following 6 h infection with both Ms_Rv1169c and the control strain Ms_Vec, Macrophages infected with Ms_Rv1169c released more LDH compared to Ms_Vec after 24 h infection and later time points (Figure 7), suggesting Ms_Rv1169c has can induce the cell death of macrophage.

### Rv1169c selectively regulates the expression of pleiotropic pro-inflammatory cytokine IL-6

**Figure 8:**
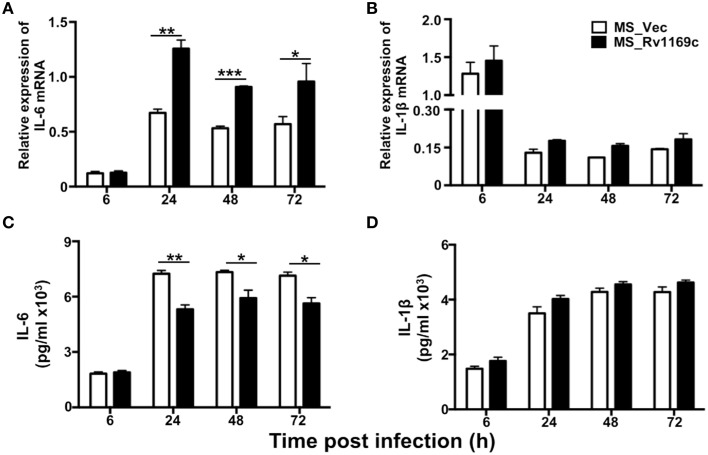
**The expression of cytokines in macrophage infected by Ms_Vec and Ms_Rv1169c**. PMA-differentiated U937 macrophages (2 × 10^6^/well/2 ml) were infected with Ms_Vec and Ms_Rv1169c strains. After 6, 24, 48, and 72 h infection, the infected macrophage were collected and the transcription of IL-6 **(A)** and IL-1β mRNA **(B)** was detected by RT-PCR. Culture supernatants were harvested, the secretion of IL-6 **(C)** and IL-1β **(D)** were detected by ELISA analysis. ^*^ *P* < 0.05, ^**^ *P* < 0.01, and ^***^ *P* < 0.001.

To explore whether Rv1169c helps *M. smegmatis* subvert the early immune responses, PMA-differentiated U937 macrophages were infected with Ms_Vec and Ms_Rv1169c for 6, 24, 48, and 72 h. The infected macrophages were collected to detect the relative expression of cytokines by RT-PCR, total RNA was extracted from the infected macrophages at different intervals (6, 24, 48, and 72 h). The supernatants were harvested for cytokines production using ELISA. Interestingly, macrophages infected with Ms_Rv1169c secreted significantly lower amounts of the pro-inflammatory cytokine IL-6 than Ms_Vec (Figure 8C), while IL-6 mRNA level mRNA of Ms_Rv1169c infected macrophages was higher than Ms_Vec (Figure 8A). These suggest that Rv1169c serves as a double-edged sword in regulation of the transcription and translation of host cytokine IL-6. The relative expression of IL-1β was induced following 6 h infection with both Ms_Rv1169c and Ms_Vec strains and their expression was decreased quickly at later time points (Figure 8B), while the secretion of IL-1β was increased after 6 h infection in both bacterial infected macrophages (Figure 8D).

## Discussion

There are increasing evidences suggesting *M. tuberculosis* PE/PPE proteins represent one of the most intriguing aspects of the *M. tuberculosis* genome (Fishbein et al., 2015). Selected members of this family have been reported to be associated with cell wall and serve as virulence factors to disturb the function of macrophage (Jha et al., 2010; Dong et al., 2012; Iantomasi et al., 2012; Tiwari et al., 2012; Thi et al., 2013). Rv1169c was found in the membrane protein fraction of *M. tuberculosis* H37Rv but absent in the culture filtrate (Malen et al., 2010). Experimental evidence for its cell wall localization remains absent. In this study, we successfully constructed the recombinant Ms_Rv1169c and confirmed Rv1169c is a cell wall associated protein.

As a predicted vaccine candidate (Narayana et al., 2007), the function of Rv1169c remains largely unknown. Rv1169c is a member of the *M. tuberculosis* PE/PPE family, also a predicted lipase lipX (http://tuberculist.com). It has been shown that PE_PGRS63 (encodes for LipY) hydrolyzes triacylglycerol stored in lipid inclusion bodies, which provides energy for the dormant *Mycobacteria* (Saxena et al., 2013). We found that a distinct cell wall fatty acid component in Ms_Rv1169c that is significantly different from Ms_Vec and wild type *M. smegmatis* strain, suggesting a potential role of Rv1169c in cell wall fatty acid metabolism. The changes in cell wall fatty acid component of recombinant Ms_Rv1169c might affect the function of mycobacterial cell wall, which is an important defense against stresses from environment. Many acid-sensitive mutants showed defect cell wall function, and also sensitive to antibiotics and other stresses (Vandal et al., 2009). We identified recombinant Ms_Rv1169c is more susceptible to surface stress SDS and acid stress compared to Ms_Vec, implicating a role of Rv1169c in stress response. Further evidence proved that Ms_Rv1169c is more sensitive to antibiotics including Rif and Nor. These results indicate that Rv1169c may increase the cell wall permeability of mycobacteria by affecting their stability.

Macrophage is the primary residence of infected *M. tuberculosis*, which sought to contain the invaded pathogens via multiple mechanisms (Leemans et al., 2005). The interaction between the *M. tuberculosis* and macrophage is crucial for the outcome of infection (Loeuillet et al., 2006). The evidence showed *M. tuberculosis* can escape from the attack of host macrophages and persist in hostile environment (Flynn and Chan, 2003), including reactive oxygen reactive nitrogen compounds, iron-deprived conditions and low pH conditions. We found recombinant Ms_Rv1169c is more sensitive to *in vitro* acid condition and SDS than Ms_Vec. Moreover, this phenotype is not correlated with increased survival within macrophage, as Rv1169 enhanced the intracellular survival of *M. smegmatis*. Pervious study has shown that necrotic cell death induced by bacterial infection is one strategy that promotes bacterial dissemination, while blocking apoptotic cell death, which serves as an innate defense against *M. tuberculosis* infection (Behar et al., 2011). It is smeared how *M. tuberculosis* operates host cell necrosis and manages its growth especially during persistent infection, although necrotic cell death commonly observed in mycobacterial lesion and granulomas is driven by bacterial factors (Dobos et al., 2000). We demonstrated that Rv1169c helps *M. smegmatis* enhance cell death with unknown mechanism. This is an important virulence mechanisms of *M. tuberculosis* appears to be their ability to induce necrosis of host immune cells, as not only Rv1169c, ESAT-6 and PE25/PPE41 have also been demonstrated to induce necrosis of immune cells via an unknown mechanism (Welin et al., 2011). The cell death induced by Rv1169c might not be apoptosis but necrosis since there is no difference in mRNA level of well-known apoptotic cell death related proteins such as bcl2 and caspase-1 (Figure S1) and cytokines TNF-α (Figure S2) and IL1β (Figure 8B) after both Ms_Rv1169c and Ms_Vec infection. This needs to be further verified since a new strategy has been recently found that IL-32-mediated apoptotic pathway is involved in controlling *M. tuberculosis* infection (Bai et al., 2015).

IL-6, a pleiotropic pro-inflammatory cytokine, is an indicator of many human chronic inflammatory diseases. Pervious studies have shown that IL-6 is crucial for the control of *Mycobacterial* infections due to its roles in producing protective Th1 immune responses against *M. tuberculosis* after subunit vaccine vaccination (Leal et al., 1999). In addition, IL-6 KO mice became more susceptible to very high doses of intravenously infected *M. tuberculosis* (Ladel et al., 1997) and lower the protective effect against *M. tuberculosis* infection (Leal et al., 1999). Moreover, other groups have proved IL-6 is critical for controlling infection in mice challenged with high doses of intravenously delivered *M. tuberculosiss* (Ladel et al., 1997; Saunders et al., 2000; Nagabhushanam et al., 2003). The depletion of IL-6 can aggravate other mycobacterial infection, such as *M. avium* (Appelberg et al., 1994). In this study, we demonstrated Rv1169c controls the expression of IL-6. This modulation is selective, since no discernable effect on IL-1β and other cytokines including IL12p40, TNF-α and IL-10 (Figure S2). However, how *M. tuberculosis* Rv1169c selectively regulates host pleiotropic pro-inflammatory cytokine IL-6 remains to be determined, as Rv1169c promotes the relative expression of IL6 mRNA while decreases the IL-6 secretion. As we noticed that Rv1169c contains a transcriptional regulator characterized helix-turn-helix motif from amino acid 88 to 109 in its C terminal. Therefore, we suspected that Rv1169c, like other regulators, might regulate the IL-6 transcription, and this might be responsible for the highly IL-6 mRNA level detected in Ms_Rv1169c infected cells. On the other hand, like other well-documented PE/PPE proteins, Rv1169c has the ability to control the secretion of cytokine, and this might be the reason for the reduced IL-6 secretion in Ms_Rv1169c infected macrophage.

## Conclusion

In summary, despite intense studies on the PE and PPE families of mycobacterial proteins, conclusive evidences for their roles remain to be found. We provide here evidence that *M. tuberculosis* Rv1169c regulates macrophage IL-6 secretion and plays an important role in cell death of macrophage. It's first reported that Rv1169c may directly or indirectly involves in fatty acid metabolism, resulting in the remarkable changes in fatty acid component of mycobacterial cell wall. Moreover, a role of Rv1169c in stability of mycobacterial cell wall integrity was confirmed as evidenced by the declined resistance of recombinant *M. smegmatis* to anti-mycobacterial factors including SDS, acid stress and antibiotics.

## Author contributions

These experiments were conceived and designed by WD and JX. Most experiments performed by WD and JZ, they contributed equally to this paper. XX and PL conducted several experiments. This paper wrote by WD and JX. All authors have read and approved the manuscript.

### Conflict of interest statement

The authors declare that the research was conducted in the absence of any commercial or financial relationships that could be construed as a potential conflict of interest.

## Abbreviations

TB: Tuberculosis
*M*.tuberculosis (Schrager and Vaccine Prevention of Sustained *Mycobacterium* tuberculosis Infection Summary, 2015): *M. smegmatis, Mycobacterium smegmatis mc^2^155*
Hyg: hygromycin
Van: vancomycin
Rif: rifampicin
INH: isoniazide
Nor: norfloxacin
SDS: Sodium Dodecyl Sulfate
IL-6: interleukin-6
IL-1β: interleukin-1β
IL-10: interleukin-10
TNF-α: tumor necrosis factor-α
LDH: lactate dehydrogenase.
