# Supplementary material for: PE11 (Rv1169c) selectively alters fatty acid components of Mycobacterium smegmatis and host cell interleukin-6 level accompanied with cell death
Source: Front Microbiol. 2015 Jun 23;6:613. doi: 10.3389/fmicb.2015.00613 (PMC4477156; doi:10.3389/fmicb.2015.00613)
Supplement: Supplementary file 1 [file DataSheet1.DOCX]

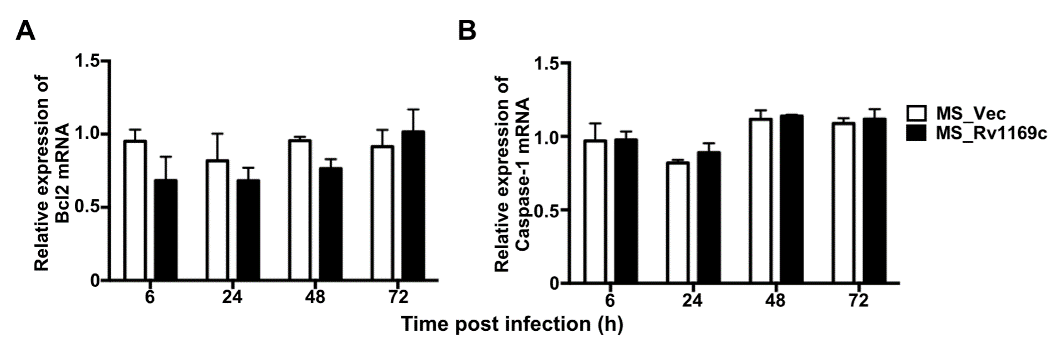


Fig s1. The relative mRNA level of Bcl2 (A) and Caspase-1 (B) in macrophage after infection with Ms_Rv1169c and Ms_Vec.


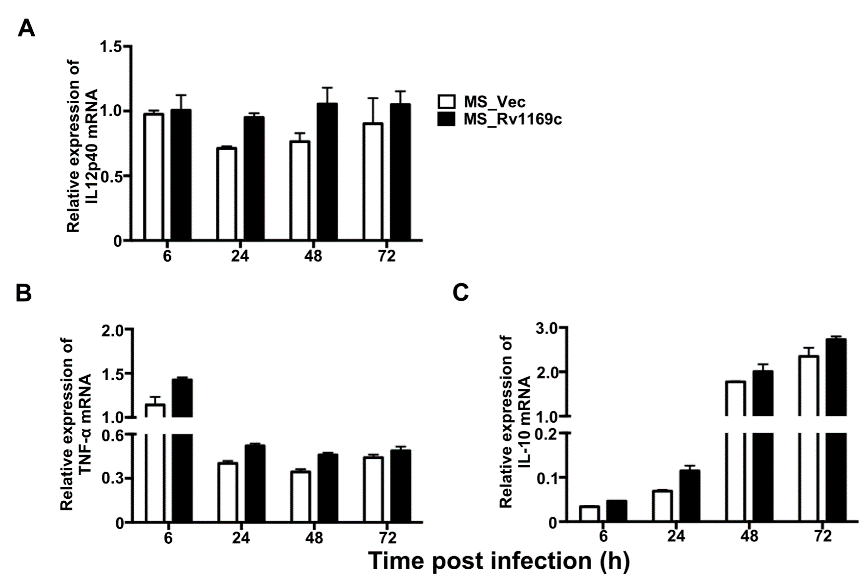


Fig s2. The relative mRNA expression of IL12p40 (A), TNF-α (B) and IL-10 (C) in macrophage after Ms_Vec and Ms_Rv1169c infection.
